# Combined effects of gender affirmation and economic hardship on vulnerability to HIV: a qualitative analysis among U.S. adult transgender women

**DOI:** 10.1186/s12889-020-08902-3

**Published:** 2020-05-26

**Authors:** Larissa Jennings Mayo-Wilson, Eric G. Benotsch, Sheila R. Grigsby, Sarah Wagner, Fatmata Timbo, Tonia Poteat, Lauretta Cathers, Ashlee N. Sawyer, Shelby A. Smout, Rick S. Zimmerman

**Affiliations:** 1 Indiana University School of Public Health, Department of Applied Health Sciences, Center for Sexual Health Promotion, 1025 E. 7th Street, Bloomington, IN 47405 USA; 2 Johns Hopkins Bloomberg School of Public Health, Department of International Health, Social and Behavioral Interventions Program, 615 N. Wolfe Street, Room E5038, Baltimore, MD 21205 USA; 3 Virginia Commonwealth University, Department of Psychology, 806 West Franklin Street, Richmond, VA 23284 USA; 4 University of Missouri – St. Louis, College of Nursing, 221 NAB South Campus, University Blvd St. Louis, St. Louis, MO 63121 USA; 5 University of North Carolina, Department of Social Medicine, CB #7240, Chapel Hill, NC 27516 USA; 6 Louisiana State University Health New Orleans School of Nursing, 1900 Gravier Street, Room 5B14, New Orleans, LA 70112 USA

**Keywords:** Transgender women, Housing, Employment, Economic, Qualitative, U.s., HIV, Minority

## Abstract

**Background:**

Transgender women (“trans women”), particularly African-American and Latina trans women, have disproportionately high prevalence of HIV in the United States (U.S.). In order to decrease gender dysphoria and overcome discrimination, trans women affirm their gender through social and medical transition, often in contexts of economic hardship and sexual risk. This study qualitatively examined how gender-affirming behaviors enhance or diminish vulnerability to HIV in light of structural and economic barriers to gender transition.

**Methods:**

We conducted individual interviews with 19 adult trans women in two U.S. cities (Richmond, VA and St. Louis, MO) who reported one or more sexual risk behaviors and recent economic hardship related to employment/income, housing, or food security. Interviews were recorded, transcribed, and analyzed using thematic content analysis.

**Results:**

The majority (74%) of trans women were racial/ethnic minorities with mean age of 26.3 years. Gender-affirming behaviors varied with 58% of trans women having legally changed their name and gender marker; 79% having initiated hormone therapy; and 11% having not initiated any medical or legal changes. None had undertaken surgical changes. Findings suggested that the process of gender transitioning resulted in both increasing and decreasing HIV risk. The high need for gender affirmation by male sex partners contributed to trans women’s exposure to sexual objectification, sexual risk behaviors, and conflicting interests in HIV prevention messaging. Loss of housing and employment due to transition along with the high costs of transition products and medical visits increased reliance on sex work and created new obstacles in accessing HIV services. Trans women experienced lower HIV risk as they acquired legal and medical transition services, reshaped interactions with sex partners, and received gender-affirming support by others, including health providers, employers, peers, and housing professionals. Sexual abstinence was viewed as a negative consequence of incomplete transition, although characterized as a period of low HIV risk.

**Conclusions:**

Structural and policy initiatives that promote safe gender transition and economic stability in trans women may play a critical role in reducing HIV in this population. Addressing the harmful pressures for U.S. trans women to conform to perceived feminine stereotypes may also serve an important role.

## Background

Given the disproportionate burden of HIV in transgender women (“trans women”), there is growing priority to understand trans women’s access to HIV prevention services and how gender-affirming behaviors may enhance or diminish HIV vulnerability [1]. Trans women are individuals whose gender identity – such as woman, trans woman, male-to-female, non-binary, or genderqueer – differs from their assigned male sex at birth [2, 3]. Trans women often affirm their gender via medical, social, and/or legal transition, which may occur concurrently or separately and at different rates and points in time [4, 5]. Medical transition can involve costly procedures such as hormone therapy, surgery (e.g. vaginoplasty, breast augmentation), or electrolysis to achieve one’s desired feminization [1, 3, 4]. Social transitioning may involve several processes, such as changing one’s dress, voice, and personal pronouns to coming out to friends and family. Trans women may also pursue legal transitions to change their name or gender marker on identification cards and records. These gender transition experiences can vary significantly with some individuals who have completed gender transition recently or years ago to those who are in the process of gender transitioning over several months or years to those who have transition plans, but have not yet begun [1].

Recent statistics show that trans women are among the most vulnerable populations to acquire HIV infection in the United States (U.S.) [6, 7]. According to the Centers for Disease Control and Prevention (CDC)‘s systematic review and meta-analysis (2006–2017) of HIV among the U.S. transgender population, laboratory-confirmed HIV prevalence in transgender individuals is 9.2%, over 30 times the prevalence in the U.S. population (0.3%) [7, 8]. HIV infection prevalence is higher among trans women (14.2%), especially trans women who are racial/ethnic minorities [8]. Approximately 44% of Black trans women are living with HIV with high HIV prevalence also among Hispanic/Latina (25.8%) trans women, compared to 6.7% of White trans women [8]. Global behavioral and biomedical strategies have additionally targeted trans women as a key population in the prevention and management of HIV given their higher reported numbers of engagement in sex work (38%), unprotected sex (38%), sex while drunk or high (37%), sex with multiple partners (42%) and sex with HIV-positive partners (20%) [8]. However, there is a paucity of data on the experiences of trans women, particularly those of color, who are at risk of, or living with, HIV [5].

HIV research that has focused on trans women has identified several barriers in access to HIV care, including pervasive mistreatment, discrimination, and violence [7]. Numerous studies have also highlighted economic inequities associated with HIV vulnerability in trans women such as income insecurity [4, 6, 9], health care costs [4, 10], housing instability [1, 4, 6, 9, 11–13], and un−/underemployment [9, 11, 12]. The unemployment rate among transgender people of color in the U.S. (20%) is four times higher than the national average (5%) [7], contributing to 29% of U.S. trans women currently living in poverty (<$14,000 annual income) [7]. Approximately one third (30%) of U.S. trans women also report having experienced homelessness in their lifetime [7].

The compounding impact of economic hardship can increase trans women’s engagement in behaviors known to increase risk to HIV, particularly during gender transition. For example, some studies have suggested that economic inequities exacerbated by incomplete insurance coverage and the need to fund medical transition may contribute in part to engagement in sex work [3, 7, 14]. Trans women who rely on sex work and who are unable to access transition-related medical care are more likely to report use of non-prescribed hormones [4], as well as alcohol and substance misuse, both associated with increased risk of HIV acquisition and transmission [5, 15, 16]. By comparison, over a third of U.S. trans women of color have not changed their name or gender marker due to costs [7, 9], and incomplete legal transition has also been found to be associated with higher reported postponement of medical care and non-prescribed injections [9]. As trans women socially transition, changing their body’s appearance and identifying new sexual partners, they may also have higher rates of condomless sex due to diminished desire to use “male” condoms [2, 17]. However, few studies have examined the intersection of economics, transition, and HIV risk among trans women, based on the voices of trans women themselves and in communities of color. Likewise, few studies have highlighted positive achievements of trans women of color in overcoming obstacles to successful transition and maintenance of their sexual health. HIV research to-date in the U.S. has largely included statistical epidemiological studies, while the limited number of HIV-related qualitative studies of trans women are often from Southeast Asia [18–21] and Latin America [22–25] or, conducted in the U.S., but conflated with a broader category of men who have sex with men [5]. Therefore, less is known about the unique experiences of ethnically diverse U.S. trans women and their specific transition and HIV care priorities. The few available qualitative studies of HIV and U.S. trans women have found that trans women of color with constrained resources seek to have their gender affirmed by others (e.g., gender affirmation) in high-risk contexts that increase their vulnerability to HIV [26–29].

This formative study aimed to provide insight into trans women’s gender transitioning experiences – challenges and successes – and to shed light on the interactive effects of gender affirmation and economic hardship on vulnerability to HIV. To this end, we also aimed to characterize economic and structural factors influencing trans women’s access to gender transitioning and HIV services.

## Methods

### Study design

This study employed a cross-sectional qualitative research design using in-depth interviews with adult trans women living in two U.S. cities, St. Louis, MO and Richmond, VA. Data from the interviews were collected from July 2018 to March 2019.

### Setting

According to the 2017 HIV Surveillance Report, Richmond, VA and St. Louis, MO ranked 30th and 48th, respectively, in number of HIV diagnoses out of 108 metropolitan statistical areas of residence in the U.S. [30]. However, despite the disproportionate burden of HIV among trans women nationally, there is limited available surveillance data of HIV within local transgender communities. The most recent HIV surveillance statistics by both the Missouri Department of Health and Senior Services (2017–2018) and the Virginia Department of Health (2016–2017) continue to conflate trans women (TW) with men who have sex with men (MSM) [31, 32]. In 2017, TW/MSM made up 74.0% of new HIV diagnoses in St. Louis, and Black/African-American individuals in the city made up 68.9% of new HIV diagnoses in 2017 compared to 23.4% White and 4.3% Hispanic individuals [31]. Black/African-American TW/MSM in Richmond/Central Region of Virginia made up 72.0% of the area’s new HIV diagnoses [32]. Our previous research uniquely among trans women in the mid-Atlantic Region found that 32% were living with HIV and 10% reported unknown HIV status [33]. Over a third (39%) of trans women were unemployed compared to 52% reporting housing instability in the last year and 62% reporting annual income <$15,000 [33, 34]. To address this gap, in both sites, there is an increasing number of publicly listed community initiatives which focus on inclusion and support of trans women, including provision of trans women-friendly housing and health care networks. However, there were considerably more established places for trans women in Richmond as compared to St. Louis. And, participation for trans women of color (e.g. Blacks, Latinas, and other races/ethnicities) in some trans women-friendly services may be limited given reports of feeling unwelcome in existing predominately White, transgender advocacy and social support groups [35].

### Eligibility and recruitment

Eligible participants included trans women aged 18 to 45 who were: (i) economically vulnerable, defined as being unemployed, employed < 19 h per week, low income (< Medicaid poverty threshold), lacking a place to stay in the last 12 months (e.g., housed by shelter, streets, hotel, or friends), or missing a meal due to lack of food or money in the last 12 months; and (ii) behaviorally at-risk for HIV, defined as reported condomless sex in the past 6 months plus an additional HIV risk factor in the past 6 months, such as transactional sex, sex while using alcohol or drugs, multiple sex partners, or one-time sex partners. We also included a subset of previously, but not currently, economically vulnerable trans women, defined as having any one of the above-mentioned economic hardships greater than 12 months ago (but not in the past year). Previously economically vulnerable trans women were included in order to provide potential insight into their experiences in mitigating any economic obstacles of gender transition and sexual health. Eligibility was determined by self-report using a computer-administered, password-protected screening tool. The screening tool obtained information on age, sex assigned at birth, current gender identity, current city residence, employment status, housing and food insecurity, recent sexual behaviors, and contact information (e.g., name, phone number, and email address). Quantitative information on education, race/ethnicity, HIV status, access to HIV information and care, sexual partners, and hormonal therapy use was also obtained in order to characterize the transition and sexual health context of enrolled trans women.

All trans women were recruited via one of two strategies: in-person or online. For in-person recruitment, a recruitment flyer was emailed to and posted and distributed on-site at community-based organizations (CBOs) (e.g., five CBOs in Richmond and five CBOs in St. Louis), each providing clinical and/or social services to trans women. Flyers were also distributed at one HIV clinic and other venues and social events frequented by trans women. The flyers were geared towards potentially-eligible trans women and included information on the purpose of the study, eligibility criteria, planned activities, and contact information of the study team. In addition to posting flyers, participating site managers were also asked to email, if applicable, the flyers to their trans women client list-serves and to refer by word-of-mouth any potentially-eligible trans women. In both cities, at least one study investigator or two graduate research assistants (RAs) then visited each participating site on scheduled recruitment days to introduce potentially-eligible trans women to the study. Snacks were provided. Interested trans women were screened for eligibility, consented, and administered the in-person interview.

For online-recruitment, we described the purpose of the research on a study-managed Facebook page. We invited any potentially-eligible trans women to complete an online screening tool via a secure web link. This web link was also circulated by email via the CBO list-serves. Potentially-eligible trans women who completed the online screening link were then invited to provide their name and contact information (e.g. email and/or phone). Those who met the eligibility criteria were contacted by the study team within 2 to 3 days to schedule an in-person interview at a mutually agreeable location, such as the CBO or a university campus meeting room near the study office. Reminders in the form of text messages, emails, or phone calls were also sent to trans women whose interviews were scheduled more than 1 day later. All trans women who completed the screening tool (regardless of eligibility status) received payment for their time, $5 in cash or via an electronic gift card in Richmond and $10 via an electronic gift card in St. Louis. Incentives varied slightly between the two study sites in response to community preferences regarding the amount and type of incentive offered. For study site selection, Richmond and St. Louis were chosen as two relatively similar sites given the need to support underserved trans women, to enhance HIV prevention services in the U.S. Midwest and mid-Atlantic regions, and to build upon prior community partnerships of the study investigators in both sites.

### Data collection

Qualitative interviews were conducted by a team of study investigators and RAs: two RAs in St. Louis and two RAs in Richmond who used a semi-structured interview guide with open-ended questions. All RAs received a five-hour training (e.g. participatory sessions with mock interviews and role play) to review the interview guide, human subjects’ guidelines, information on the transgender community, the study protocol, and qualitative interviewing techniques. The study investigators developed the interview guide with contributions from each of the two community advisory boards (CAB) in St. Louis and Richmond. The CABs included trans women, trans men, and cisgender persons who were members of local non-governmental organizations (NGOs) providing medical, social, and/or mental health services to transgender individuals and/or persons living with HIV. The primary role of the CABs was to review and provide feedback on all study instruments and processes, including the consent and screening tools, and to assist in recruitment. Following review from the CABs, the final interview guide asked enrolled trans women to describe their gender transition process, including what services or products they had used or planned to use to support transition and any prior or anticipated transition costs. Transition products included any items or goods that were used to facilitate gender transitioning, such as bra pads, make-up, clothing, accessories, hair removal products, hormones, voice lesson materials, etc. We also asked trans women to describe experiences obtaining employment and housing before, during, and/or after gender transition – and how they responded to any gender-related discrimination, including suggestions for ways to improve financial outcomes of trans women in the future. Trans women were additionally encouraged to discuss their access to HIV prevention services relating to testing and pre-exposure prophylaxis (PrEP) and circumstances (if applicable) leading to unprotected and/or transactional sex. A final set of questions asked trans women to provide feedback on potential HIV and microeconomic interventions that were tailored to U.S. trans women. However, given the volume of responses, intervention feedback data are presented in a separate, forthcoming manuscript. Interviews were conducted in English. They lasted approximately 60 to 90 min and were audio-recorded. All interview trans women were given an additional $50 in cash immediately after the interview.

### Sample size

The target sample size was 20 trans women: 10 in St. Louis and 10 in Richmond. A small qualitative sample was chosen given the exploratory aims of the study and to reduce research burden within vulnerable transgender populations. To achieve saturation with a relatively small sample, we used a purposive homogeneous sampling strategy of trans women experiencing economic hardship and who reported recent sexual risk-taking. Homogeneous sampling strategies select a sub-group of individuals with common circumstances in order to yield a more focused analysis [36, 37]. We also used a prolonged interviewing period (up to 1.5 h) with several in-depth questions to obtain diverse opinions among the limited number of individuals [38].

### Analysis

All interviews were transcribed and included in the analysis. We used an integrated content analysis methodology to categorize verbal data based on emergent themes within previously identified topics [39, 40] relating to gender transition, housing, employment, and access to HIV prevention services. To achieve this, a trained graduate RA developed a preliminary codebook based on a close reading and coding test of the first eight available transcripts. Comments and edits to the preliminary codebook were then obtained from the study investigators based on readings of the transcripts and discussions of early findings. Next, the RA used a finalized codebook to code available and incoming transcripts over the period of October 2018 to February 2019 using ATLAS.ti, a qualitative data analysis software (Version 8.3.1, https://atlasti.com, Corvallis, OR, USA). During the coding process, the RA and a study co-investigator additionally prepared analytical memos to note potential themes, including interpretations and relations between codes. These notes were then reviewed and further analyzed by the larger study team (from St. Louis and Richmond) during a two-day data synthesis workshop in March 2019. Based on these discussions, the remaining transcripts were coded and added to the analytical memos (April to May 2019). During this process, we also extracted quotations that illustrated key themes. To provide a sense of the predominance of some themes relative to others, we documented the general number of trans women who discussed each theme. In addition, to characterize trans women’s transition stage, we developed an Excel-based data extraction form to query each transcript for information on trans women’s use of “female” dress (e.g., clothing, makeup, hair), hormone therapy, legal change of name, legal change of gender marker, or transfeminine surgery. We then tabulated the number and frequency of types of transition in the study sample. As a final step, a subset of quotations was selected for presentation in this manuscript and labeled by age and employment status (e.g., full-time (FT), part-time (PT), or unemployed). Given the small sample and potentially increased familiarity within transgender communities, race, HIV status, site, and transition stage were excluded from quotation labels to safeguard trans women’s anonymity.

### Ethics

This study obtained ethical approval from the Virginia Commonwealth University (VCU) Office of Research and Innovation Institutional Review Board (IRB) (#HM20011245) and the University of Missouri – St. Louis (UMSLM) IRB (#1179371–2). Oral informed consent was approved by both IRBs as the signature requirement would have been the only record linking the subject and the research, and a possible risk would have been potential harm resulting from a breach of confidentiality. Oral informed consent was obtained from each trans woman prior to the start of the interview.

## Results

### Sample characteristics

Table 1 describes the demographic and economic characteristics of the study’s participants. A total of 19 trans women were enrolled in the study: 11 trans women in Richmond (58%) and 8 trans women in St. Louis (42%) (Table 1). Data collection was ended at sample size *n* = 19 due to reaching information saturation. The mean age of participating trans women was 26.3 years, ranging from 18 to 50 years. The majority of the sample were trans women of color (74%, *n* = 14), including 63% (*n* = 12) African-American, 5% (n = 1) Latino/Hispanic, and 5% (n = 1) Asian compared to 26% (*n* = 5) who were White. Highest level of education varied across the sample. Twenty-one percent (21%, *n* = 4) had primary education only compared to 42% (*n* = 8) who had completed high school and/or the equivalent. A quarter (26%, n = 5) of the sample had some college training compared to 11% (*n* = 2) who had graduated from a college/university. Two trans women (11%, n = 2) reported being previously but not currently economically vulnerable. Approximately one third (32%, *n* = 6) of trans women were unemployed, and among those employed (*n* = 13), most (77%, *n* = 10) were under-employed with part-time assignments. Housing insecurity (74%, n = 14) in the past year was also high.

**Table 1:** Sample demographic and economic characteristics by site and total

	Site		Total
	Richmond	St. Louis	
Number of enrollees	11	8	19
Percentage of total sample	58%	42%	100%
*Demographic characteristics*			
Mean age in years	24.8	28.3	26.3
Age range (min, max)	20–30	18–50	18–50
Highest level of education			
Primary	0	50% (*n* = 4)	21% (*n* = 4)
High school diploma	64% (*n* = 7)	12% (*n* = 1)	42% (*n* = 8)
Some college	36% (*n* = 4)	12% (*n* = 1)	26% (*n* = 5)
College graduate	0	25% (*n* = 2)	11% (*n* = 2)
Race/Ethnicity			
African-American	73% (*n* = 8)	50% (*n* = 4)	63% (*n* = 12)
Latino/Hispanic	9% (*n* = 1)	0	5% (*n* = 1)
Asian	0	12% (*n* = 1)	5% (*n* = 1)
White	18% (*n* = 2)	38% (*n* = 3)	26% (*n* = 5)
*Economic characteristics*			
Previously but not currently economically vulnerable			
Yes	9% (*n* = 1)	12% (*n* = 1)	11% (*n* = 2)
No	91% (*n* = 10)	88% (*n* = 7)	89% (*n* = 17)
Currently employed			
Yes	64% (n = 7)	75% (n = 6)	68% (n = 13)
No	36% (n = 4)	25% (n = 2)	32% (*n* = 6)
Full-time employed^a^			
Yes	43% (*n* = 3)	0	23% (*n* = 3)
No	57% (*n* = 4)	100% (*n* = 6)	77% (*n* = 10)
Lacked housing in past year			
Yes	73% (*n* = 8)	75% (*n* = 6)	74% (*n* = 14)
No	27% (*n* = 3)	25% (*n* = 2)	26% (*n* = 5)

[a] Excludes trans women who were unemployed

Table 2 describes the HIV and gender transition characteristics of the sample. Thirty-seven percent (37%, *n* = 7) of trans women reported being HIV-positive with 2 trans women (11%) reporting unknown HIV status (Table 2). All trans women reported recent condomless sex (100%, *n* = 19). Two-thirds (63%, *n* = 12) of the sample self-identified as “trans woman” compared to 37% (n = 7) who preferred to be identified as “woman” or “female”. Use of hormone therapy was high (79%, *n* = 15), although changes to one’s legal name (42%, *n* = 8) and official gender marker (42%, n = 8) was lower. Two trans women (*n* = 2, 11%) reported having initiated no legal or hormonal transitions. No trans women (*n* = 0) had received surgical procedures to affirm their gender.

**Table 2:** Sample HIV and transition characteristics by site and total

	Site		Total
	Richmond	St. Louis	
Number of enrollees	11	8	19
Percentage of total sample	58%	42%	100%
*HIV Characteristics*			
HIV Status			
Positive	27% (*n* = 3)	50% (*n* = 4)	37% (*n* = 7)
Negative	64% (*n* = 7)	38% (*n* = 3)	53% (*n* = 10)
Unknown	9% (*n* = 1)	12% (*n* = 1)	11% (*n* = 2)
Condomless sex in last 6 months			
Yes	100% (*n* = 11)	100% (*n* = 8)	100% (*n* = 19)
No	0	0	0
*Gender Transition*			
Self-reported gender identity			
Trans woman	64% (*n* = 7)	62% (*n* = 5)	63% (*n* = 12)
Woman/female	36% (*n* = 4)	38% (*n* = 3)	37% (*n* = 7)
Man/male	0	0	0
Legally changed name			
Yes	55% (*n* = 6)	25% (*n* = 2)	42% (*n* = 8)
No	45% (*n* = 5)	75% (*n* = 6)	58% (*n* = 11)
Legally changed gender marker			
Yes	45% (*n* = 5)	38% (*n* = 3)	42% (*n* = 8)
No	55% (*n* = 6)	62% (*n* = 5)	58% (*n* = 11)
Initiated hormone therapy			
Yes	82% (*n* = 9)	75% (*n* = 6)	79% (*n* = 15)
No	18% (*n* = 2)	25% (*n* = 2)	21% (*n* = 4)
Dressed as “female” always or occasionally^b^			
Yes	100% (*n* = 11)	100% (*n* = 11)	100% (*n* = 11)
No	0	0	0
Had any surgical procedures			
Yes	0	0	0
No	100% (*n* = 11)	100% (*n* = 11)	100% (*n* = 11)
Reported none of the above hormonal, surgical or legal changes			
Yes	9% (*n* = 1)	12% (*n* = 1)	11% (*n* = 2)
No	91% (*n* = 10)	88% (*n* = 7)	89% (*n* = 17)

[a] Includes reported current use of pre-exposure prophylaxis (PreP) or antiretroviral therapy (ART) for HIV- or HIV+ trans women, respectively; [b] Includes wearing “women’s” clothing, make-up, and hair/wigs

### Emergent themes

A total of 19 in-depth interviews were conducted with 19 trans women (e.g., one interview per trans woman). Table 3 lists the emergent themes described by trans women across four previously-identified study topics: gender transition (6 themes), employment (6 themes), housing (2 themes), and HIV-related services (4 themes). A summary of exemplary quotations follows below.

**Table 3:** Summary of interviews’ emergent themes and relative frequency of discussion by topic

Study topic	Relative frequency of discussion^**a,b**^	Emergent themes
Gender transition	****	Transition occurs in stages but does not define womanhood
	******	Financial barriers to medical transitioning
	****	Use of lower-cost and non-prescribed medications
	**	Taking on only small transition expenses
	**	Waiting to “come out” until transition is completed
	******	Limited access to gender affirming services
Employment	*	Termination due to disclosed transgender identity
	******	No transition or full transition is most protective against job discrimination
	******	Sex exchange as source of employment while gender transitioning
	****	Challenges with name change and name preferences at work
	******	Need for trans-sensitivity training for local employers
	****	Higher pre-transition earning power
Housing	**	Useful to have trans-supportive housing
	**	Difficulty accessing housing due to transgender identity
HIV services	******	Linking transition services to HIV testing/care
	****	Concerns for trans discrimination by health care system
	****	Discussing HIV prevention with sex partners
	****	Effect of transition on sex partner accessibility and relationship

[a] Based on n = 19 trans women; [b] Relative frequency of responses are denoted by: * discussed by < 20% trans women; ** discussed by 20 to 35% trans women; ****discussed by 35 to 50% trans women; ****** discussed by > 50% of trans women

### Gender transitioning

#### Transition occurs in stages but does not define womanhood

Transition experiences described by trans women were diverse and complex. A few trans women reported below being at the early stage of starting or considering whether to start hormone therapies or having recently identified new trans women social groups. Many trans women stated that they were satisfied with their transition status, although they had long-term plans to obtain surgery. In contrast, others described having achieved full transition based on an inherent decision or in response to acquiring their desired physical and legal changes without surgery or medications.

“Most of the transitioning I’ve done has been, like, social. I was able to surround myself with more trans people, and specifically more trans women. … It’s mainly just been, like, clothes and lipstick and stuff. … I might start taking estrogen in the future, but that’s probably not going to be for a while.” – Age 20, employed FT.

“I’m not going to have surgery. And the main reason I’m not is because I wasn’t created to be a woman. I’m a woman, but I wasn’t created to have a vagina. If God wanted me to have a vagina, I would have a vagina. But obviously he wanted me to be a woman or pretty enough to be a woman because I am with no surgeries, no hormones, none of that.” – Age 34, unemployed.

“I personally would like to get a sex change. I mean I still have the genitals that I was born with at birth. And, I would want that removed. … My goal is to have it done at least by the age of forty. So that gets me about, like, ten years to get everything in order.” – Age 30, employed FT.

“I go by female. Actually, I’m fully trans. My doctor gives me hormones, but I feel like the hormones I’m being prescribed are not really doing anything for me anymore. I’m not really seeing much more of a transition. I just feel like I’m in a standstill, but they say you still are transitioning … To me, I’m not seeing it.” – Age 24, unemployed.

“I’m quote-unquote ‘supposed to be’ not a woman, but I am to me! I’m more woman than a lot of women out here. Just from retrospect of things that I have done or am doing. Like I know a lot of women and girls out here that do not portray themselves as a woman. Like you have a vagina girl, but you’re not a woman.” – Age 24, unemployed.

#### Financial barriers to medical transitioning

Although experiences varied among trans women, there was consensus regarding the high expense of medical transitioning. For example, one trans woman who was uninsured described below high out-of-pocket costs for hormone medications and trans-related doctor’s visits. Two trans women described the challenge of foregoing hormone therapy as a result of an insurance denial or due to inadequate personal funds in a given month. In a few cases, transition therapies were discontinued for several months or years due to financial constraints.

“Hormones have been very expensive. The injectables are not covered by insurance and are definitely better both in their efficacy and their long-term health. So, I pay out of pocket every two months or so. I don’t have any insurance right now, so I pay full price. Almost all of my blood tests and doctor’s appointments have not been covered by anyone for any reason.” – Age 25, employed PT.

“I asked her [the doctor] if I could get this shot or whatever. But, there’s no money for it. And, I want to get my breasts done. That’s all I really want is those two things. But every time I look into Medicare, they always saying there is no funding for that. And I have hospital bills. So, it’s hard. Now my credit is shot.” – Age 24, unemployed.

“It’s difficult. It’s like, ‘Oh my, God, fifty dollars.’ I’ve known that to cause some people to de-transition because they don’t have it. They can’t find work. And, so, fifty dollars to just give away for some pills and medication is like a ‘Do I really wanna do this?’ type of situation. I’ve had one of those moments before actually. I’m like, ‘I don’t have fifty dollars this month. I’m not gonna be able to take my medicine this month.’ It’s difficult. I would just say that it’s difficult.” – Age 23, unemployed.

“I got sick for a little while, and I wasn’t able to take my hormones … So, I stopped taking [them] for a little while. There was a stretch a little over a year ago too where I just wasn’t able to afford it.” – Age 27, employed PT.

#### Use of lower-cost and non-prescribed medications

To counter high costs associated with gender transitioning, many trans women described lower-cost strategies, such as eating high-estrogen foods to enhance the development of more “feminine” patterns of hair, fat, and muscle. Avoiding costly doctor visits was commonly reported, including using non-prescribed hormone pills from friends or from online. Trans women described concerns regarding the safety of these methods, but felt they were the most cost-effective alternative. As stated by one trans woman below, relying on one’s “home girl” for medications was also viewed as easier than navigating the health care system.

“I have been doing a diet which I read is like a cheaper way of just flooding the body with estrogen. I don’t know if it works, but it definitely has made me feel a little better. … What’s involved in the estrogen heavy diet? Soy. A lot. Like soy products are really high in estrogen. … It’s known to develop like breast growth. And I’ve … noticed like physical changes that are pretty subtle, but they’re noticeable to me.” – Age 26, employed FT.

“I think one out of fifteen trans probably goes to the doctor. No, I’m lying. But probably like three out of fifteen. That’s being serious. Like [three] would be going to a doctor. Like people that you know be like, ‘Why? I don’t go to no doctor!’ Like my home girl, I call her out. She sent them to my house.” – Age 26, unemployed.

“I didn’t know why I was tired and stuff back then, you know? All I knew was I was just popping pills. I just knew the name that a person was telling me it was. … My name was not on the prescription, so you don’t get no direction or nothing like that. You just get what a person sent you. So, yeah. Like doing it in the back-alley way definitely unsafe.” – Age 26, unemployed.

“A friend told me where I could get them online. I didn’t even know what they was! It was just something that everyone was doing. I know that they said that it would help me transition and get them because it takes a while in order for me to be seen by a physician. Because they had these guidelines where they wanted you to do counseling, and that wasn’t something I was interested in.” – Age 30, employed FT

#### Taking on only small transition expenses

A few trans women highlighted the importance of using inexpensive transition products, such as make-up, bra pads, or second-hand clothing as part of their gender expression. These were seen as necessary confidence boosts.

“Aside from quite a lot of makeup, you know obviously I changed my whole wardrobe. … Most of the wardrobe actually came from friends and family members and clothing swaps and stuff like that. I’ve definitely like probably spent a couple hundred bucks on clothes, but not nearly as much as I probably would have otherwise.” – Age 25, employed PT.

“I used to stuff my bra before like my hormone boobs came in. So yeah, I would like to use inserts from like Walmart and stuff [for] only like $12. So, no, those wasn’t expensive. And … I think that’s good because … somebody that wants to transition, if they don’t have those, they might feel really bad and not even feel like they’re meant to transition. But that little bit of help … just putting a wig on is gonna give them that boost of confidence and [make them] feel like they can go up to the world and feel more real or more confident.” – Age 26, unemployed.

#### Waiting to come out until transition is completed

Gender transition was also described as a vulnerable period in which trans women were no longer presenting as “male,” but had also not yet achieved their desired “feminine” appearance. Several trans women described preferring to wait to disclose their gender identity until their transition was complete, as a means of both enjoying the process and avoiding negative feedback. As one trans woman described below, waiting until one was ready could entail forgoing or postponing employment, as well as working in a more women-centric workplace.

“If you’re not ready or you’re not fully transitioned, just wait so you don’t have to go through all that negativity. I mean you’ll get the job. But, they’re gonna be like, ‘Oh, sir?’ I mean you’re gonna have to go through that backlash. They’re going to be calling you sir. Just negativity. Who wants to go through that?” – Age 27, employed PT.

“Let’s say … you’re at work and you work at this job for so long. But now, you’re starting to transition. Like you don’t wanna do that and people start asking questions. So, I think a lot of people do that [e.g., wait] just to transition in peace, you know? Just to be out of the way and not have a lot of attention focused on you when you’re going through something, you know – mentally and physically changing dramatically, you know?” – Age 26, unemployed.

“I don’t know. What I’m saying is when I go back full on, I’m gonna be like in a feminine job. Like makeup, cosmetics, [and] stuff like that. Nothing [in a] factory where I have to be around a lot of men. Because you put yourself in that situation.” – Age 27, employed PT.

#### Limited access to gender-affirming services

Many trans women described their frustrations with the limited number of services and trans-focused organizations available to them during transition. As one trans woman described, there was only one clinic offering transgender hormone therapy and few available online. Two other trans women lamented the lack of surgical sites for trans women in their areas compared to larger metropolitan areas, and the repeated attempt of local providers to refer them to organizations for men.

“Anytime somebody asks me, the only place I can point them to is [name of organization] ‘cause that’s all I know, you know? But, if you’re like a hairdresser, you can point people to like a thousand hairdressers. You can like google them, and they pop right up. But hormone therapy, no. It’s hard. You’re digging for gold almost.” – Age 26, unemployed.

“It’s because of the way our city is. Our LGBT community needs to really step up a little bit more the trans community services. I have no surgical work done yet. Everything I have is through hormones and all of that. But I do know in different cities, they offer services to help you get your ambiance. They help you with your transition and to get all your hormones.” – Age 27, employed PT.

“You know, people treat us like we’re nothing. So, we look to everybody else. It’s not a lot of organizations that we can look to. Because everybody sends us to the male organizations. That is the problem. We have no organizations.” – Age 50, employed PT.

### Employment

#### Termination due to disclosed transgender identity

Dissatisfaction with one’s employment status was a common theme during interviews. A few trans women indicated that they were terminated once their trans identity was discovered. As one trans woman describes below, her supervisor saw her outside of work in women’s clothing and fired her the next day. Another trans woman mentioned losing a skilled job while experiencing transition and bereavement.

“One day, a guy was working. I picked up my check dressed like I usually do when I’m not working. And he hadn’t seen me like that. And he gave me this look. Then the next day, I got a message trip. The chef I love – a great guy who was always nice to me – he was like, ‘Hey! So [name] took you off the schedule. Sorry. You can use me as a reference’. He didn’t give me a reason. So, I knew the reason.” – Age 26, employed FT.

“It’s hard when you lose everything. You lose your wife. You lose your family. You lose your income because of who you wanna be.” – Age 50, employed PT.

#### No transition or full transition is most protective against job discrimination

Avoiding termination or abuse on the job was a key concern among trans women. A few mentioned achieving this by presenting as male and not initiating any form of transition. However, some trans women felt that full physical transition (as described below), including having completed legal name and gender marker changes, was necessary to be accepted by employers or clients – particularly in terms of safety and job security. “Passing” as a woman, in this sense, was viewed as providing trans women an advantage of respect and employability.

“I’m still not comfortable with being open in the workplace just yet. For the most part, I’ve been just going out in guy mode. It’s been alright because of that. I’m not sure whether or not it would be harder to get a job if I was out, but I feel like it would. … I don’t want to get fired. I mean they don’t know, so it hasn’t caused any problems. They’re cool with me.” – Age 20, employed PT.

“At the time I was working as a driver. I like never presented particularly male. I’ve always had long hair … and soft features. So, long before I came out, I was getting she’d like half the time. At this one fair (e.g., ride), by the end of it, I felt like I’m actually safer if I present as a woman than this, you know, androgynous questions closet case. So yeah. I definitely think there were a number of things including my own safety that coming out was the best way to address.” – Age 25, employed PT.

“The current job I have, I was already out. And, people already knew about me. And, I did tell my job everything too. Or, they would tell the people just so they would have a heads up. I had all of my name documents and gender marker changed also on my ID … , and [brought] all that to my current job just so they would know and everything, just in case.” – Age 18, unemployed.

“I feel like I probably don’t have it as bad because I can pass. So, it definitely is an advantage – being able to pass in the work world. Because when they can identify you, those are typically the women that get the most disrespect.” – Age 21, employed PT

#### Sex exchange as source of employment while gender transitioning

However, full gender transition or not gender transitioning was not an option for several trans women. Instead, they described quitting or losing their jobs and relying on sex work for a few months or a few years as a much-needed source of income during the early stages of gender transitioning. This period was when they first began to present as female or when they were initiating hormone therapies. As one trans woman notes below, sex work was described as giving trans women peace of mind to transition anonymously, while continuing to pay for desired gender-affirming services.

“I definitely did it. Just like I said, so I could, you know, transition in peace. I didn’t want people to see me like cute this day and then just a dramatic change or whatever. And then, you don’t even know mentally like what you project to the world. So, it’s just better to get yourself together I guess, and still make your money, you know?” – Age 26, unemployed.

“We’re desirable to a lot of people. People found it hard to hold the job down just because … random people might have horrible opinions on trans, and it can be really isolating to come out. Sometimes sex is the only option because people want to, and it’s something most people can do.” – Age 26, employed FT.

“I started noticing that my cousin was just getting paid to have sex with people. So, I’m like ‘Okay, I want to do that!’ So, I did it for a good three years. Without prostituting, I wouldn’t have made it. Like, I wouldn’t have been able to pay my cousin for staying with her. Wouldn’t have been able to keep my phone on. Wouldn’t have been able to get my hair done. Couldn’t get my nails done. So, yeah.” – Age 28, employed PT

#### Challenges with name change and name preferences at work

Other employment challenges during transition were being misgendered or feeling dysphoria when addressed at work by a pronoun or name that didn’t reflect their gender identity. As described by one trans woman below, this most commonly occurred among trans women who had not yet acquired legal documents for their new name or gender marker. On the other hand, one trans woman described how her workplace used her chosen name and masked her official name in employment documents.

“On one of my first jobs, when I first transitioned, my name wasn’t switched over. She told me … that at the call center I was going to have to use my original name. And I’m like, ‘Can I just use the first letter … plus my last name?’ And she’s like, ‘No. You need to say it clear. So, that kind of was a problem. I guess if people don’t have their information switched over, that is a problem in the jobs.” – Age 26, unemployed.

“My job treats me very well. My name is so and so on the paper. It’s so and so on my name tag. But once I get the [pay] check you know what that means. But nobody in my job besides my manager probably knows my real name because … on my check, nobody sees it but me. But everything else, it says my girl name.” – Age 34, employed PT.

#### Need for trans-sensitivity training for local employers

Despite a few positive experiences, the majority of trans women indicated that more efforts were needed to train employers to be sensitive to the needs of trans individuals and to create more transgender inclusive workplaces. Many trans women felt they should be viewed simply as an employee. It was important also to be hired for their skills and not for political or diversity reasons. As shown below, one trans woman launched a petition to raise awareness of trans women with her employer.

“Sometimes I feel like, ‘What could I do?’ … I guess it would just have to be like a statewide sensitivity training. But I don’t think it’s even like a sensitivity thing. I think it’s just like common decency because we’re all working.” – Age 26, employed FT.

“I guess they will hire some trans just to be like, ‘We’re not discriminating!’, or something like that. But, it’s still like when you’re hiring them, still treat them as family, you know? Don’t just throw somebody in the mix just to save your own butt. Actually educate yourself and take the time to care for a [trans] person basically.” – Age 26, unemployed.

“When I got the job, … I got them to change their whole bylaw because I felt uncomfortable having to go to the male’s bathroom. Definitely with me having breasts and … putting me in danger … I had them either make a unisex bathroom or I used the female’s bathroom. [And], I had a petition signed. Now it’s easier for trans women at that job.” – Age 27, employed PT.

#### Higher pre-transition earning power

Trans women also felt that more should be done to address income disparities between cis and trans individuals, including the wage gap between men and women. Some trans women noted that their salaries had dropped substantially as a result of new employment gender discrimination.

“Before when I was presenting male, I found it very easy to go into an interview and just sound like what you’re supposed to sound like. And that would get me the job most of the time. And, I made a lot more money. … At the most when I was presenting male, I made $45,000 a year. I haven’t even come close to that since then.” – Age 25, employed PT.

### Housing

#### Useful to have trans-supportive housing

Trans women in this study reported fewer vulnerabilities with housing as many lived with relatives, intimate partners, or friends. There was a general relief among trans women that living in a trans-friendly environment protected them from harmful practices. Supportive housing included shared homes/rooms and landlords who did not inquire about gender identity or who used chosen names and pronouns. A few trans women reported avoiding using their name on the lease or staying with relatives to avoid negative housing experiences. However, these strategies in some ways restricted their autonomy.

“The place that I’m staying now, my rent-lady, she is really nice. And I don’t think she knows I’m transgender. I can’t say if she do or she don’t. We never really talk about that. But she’s a really sweet lady, so.” – Age 24, unemployed.

“When I first moved in, there were little cards hanging on the doors with our names on them. And, mine had my deadname on it because I don’t use my real name for anything official here. So, when my RA found out about it, they were like, ‘Well, do you want us to change your name on the card for you?’ And they did! And, that was it. So that was a positive experience.” – Age 34, unemployed.

“Recently, I’ve been lucky to have friends who are the ones who put their names on the lease. Like right now I live with my partner and some roommates, and I am not on the lease. I know that if I was to get a note from a landlord, they would use just the government name which hurts a lot of trans people.” – Age 50, employed PT.

#### Difficulty accessing housing due to transgender identity

However, safe housing particularly during or after gender transition, was a significant challenge for some trans women. As described by three trans women below, challenges included unlawful rental exclusions, evictions, and housing requirements that trans women stay at men’s shelters. There was also concern for lack of city-appropriated funding for housing of trans women.

“The manager said, ‘No … we’re going to refuse to rent to people that aren’t of our nature because it’s going to cause too much commotion in our building. We feel like it would be a greater risk to the people in our community. So, we’re going to refuse your application.”– Age 50, employed PT.

“With my Dad, it was like I really never got any peace. ...I couldn’t have a significant other or someone I’m interested in. They couldn’t come over, you know. That was definitely difficult. And then, recently I was staying with a friend, and he just put me out. Like on the spot. Like, ‘You got to leave. I’m sorry.’ And, I’m just like, you know? I just feel like if I was a regular girl …” – Age 21, employed PT.

“There are stipulations with that because it’s a woman’s shelter. I had a friend that I had to call for. And they said, ‘Oh, no. She can’t stay here because she’s trans.” And, I’m like, ‘Trans equals woman’. It’s like, she’s transitioned to a woman, but they actually made her call a men’s shelter. And, I called the head people, and I explained to them, like, she is a woman now. Like, the men in there could do something to her.” – Age 24, unemployed.

“If you look at our budget, you have places for gays, lesbians, [and] heterosexuals. … But you have nothing for transgenders. You have nothing for … you know, people with different parts. For temporary housing, I think that we should be able to have a budget. There should be a budget in this city just like there is for other specialty need people. There’s no reason why we’re not in the budget.”– Age 50, employed PT.

### HIV services

#### Linking transition services to HIV testing/care

There were mixed feelings regarding access to gender-affirming services and HIV prevention and care clinics. On the one hand, several trans women felt that HIV clinics were not sufficiently equipped to care for trans women, lacked transgender clinicians, and unfairly characterized the magnitude of HIV in trans communities. As one trans woman indicated below, such shortcomings were viewed as exacerbating existing vulnerabilities for trans women who were “never reached out to”.

“I’m going to say this. What’s the HIV committed with? They mainly focus on just that. They don’t focus on us transitioning. … I do understand that their biggest focus is getting your health under control. … But at the same time, we have to have a separate service because what we do for the LGBTQ community for HIV versus the LGBTQ just for trans are two different things.” – Age 27, employed PT.

“Like, the commercials and stuff about HIV? We’re like one of the first people that they put on these commercials. And, it’s just like not every trans girl is walking around with HIV … I’m HIV negative. I’m like, it never fails. We’re always the people that they put on there.” – Age 23, unemployed.

“I don’t think that it’s very trans woman focused. A lot of it is, frankly, ran by older, white, cis, gay men who lived through the AIDS epidemic. … But also, a lot of trans women died, and they’re still dying from HIV. It’s not very accessible unless you reach out. Gay men are very reached out to in very gay men centric spaces. But trans women are never reached out to. It’s like, well if you need help, come find us.” – Age 18, employed PT.

“We need more trans counselors. We can’t just have a male or female as a counselor because they won’t understand our needs and what we’re dealing with. I’m really looking at it in terms of like [what’s] sensitive or appropriate for trans folks – like actually having other trans people there to handle the testing, to be there to counsel, and to be present.” – Age 27, employed PT.

In contrast, several trans women described their experiences of testing for HIV while obtaining hormones at a transgender clinic or accessing prescribed hormones from their HIV/ART care provider. The integration of these services was viewed as enabling trans women to protect their sexual health during transition.

“If I wouldn’t been getting hormones from a doctor, I wouldn’t have gotten tested, and I would have not known my status. You get what I’m saying? So, that is like wonderful …” – Age 26, unemployed.

“Sometimes through my [HIV] case manager, she’ll buy hormones for me. And when she buys them, she gets the sixty-day dosage … I go and just pick it up. So, that’s good. But she can’t always get the hormones for me. It’s like a hit and miss. Like, I’m getting ready to run out actually. … If she can’t [get them], then I’m gonna have to figure it out on my own.” – Age 21, employed PT.

#### Concerns for trans discrimination by health care system

However, some trans women discussed barriers to obtaining respectful and inclusive HIV and/or transgender care as a result of misgendering or lacking a legal name or gender marker change.

“My provider was physically queer and that helped. Any time his paperwork used unnecessarily gendered language … , he would either mentally correct them or … apologize and be like, ‘You don’t have to answer that.’ There are a number of places that still insist on if I have sex with a trans woman, that that’s a male having sex with a male. But my individual doctor is great. But, the clinic at which he works is not. They still detonate every time. And it’s legally changed! … I’ve been told repeatedly that their system won’t allow for me to be listed as female.” – Age 25, employed PT.

“And they tell me, “We have to call you what’s on your medical records until we get that name changed.’ And that’s what I’m trying to change. That’s what my biggest issue is. It’s about hormones. It’s about name change.” – Age 50, employed PT.

#### Discussing HIV prevention with sex partners

Trans women’s views on discussions of HIV prevention with their sex partners was variable. Some considered themselves as HIV prevention advocates, acquired condoms, and encouraged partners to test for HIV and take pre-exposure prophylaxis as needed. In contrast, other trans women expressed uncertainty regarding their risk for HIV given their choice of sex partners, their preference for oral sex, and discomfort with the sex characteristics of their bodies.

“My partner – he’s not positive. So, I make sure that he’s on PrEP as well. … I was actually mad when I found out about PrEP because I was like, ‘What?!’ So, I tell everybody this. ‘Look! It’s not even about the HIV status. Ok? Go to your doctor. If you don’t want HIV, go get PrEP. PrEP is … your vitamin that gets you not to have HIV, Ok?” – Age 50, employed PT.

“I often take advantage of the free condoms around. … But it’s not something that I worry about too much, simply because I have not slept with any men, and I don’t really plan to. It’s not completely off the table, but I don’t really plan to. … For the most part like I’m having like oral sex with women, and so it’s not an issue.” – Age 25, employed PT.

“I am a very feminine, heterosexual woman who still has a penis. There aren’t a whole lot of men that I would wanna have in my life [who are also] jumping to have me in their lives. So, sex-wise there’s nothing going on. I don’t even need to think about STDs because it’s not even possible. I wish it was possible, but it’s just not. … With this body of mine, I don’t even know how I have sex right now.” – Age 27, employed PT.

#### Effect of transition on sex partner accessibility and relationships

Trans women also described new sexual pressures and vulnerabilities primarily from cis men as a result of their transition. These new pressures involved sex while high to get into a woman’s mindset and appeal to men’s “feminine” preferences as well as sex in exchange for money with men who were not interested in dating. One trans woman described below losing a sex partner when she came out and efforts to find new sex partners as a result. Another trans woman stated that gender transition had resulted in new exposures to sexual violence during encounters with cis men.

“You have to look a part. You can’t be looking like a man in a wig. You have to look like a woman. Because some of these men want women. They want to feel the atmosphere of a woman. So, you be on drugs all the time. I’ve been on all types of drugs- coke, pills. Because it’s a mind thing. It’s a mental state.” – Age 34, unemployed.

“When we on the phone, and I tell them I’m a transgender, it goes from me getting to know them, getting that they want to date me, take me out, [and] show me nice things … to I want to have sex. So, back in my mind, [I say], ‘You want to have sex? I want to be paid for my time.’ It’s not basically that I’m prostituting. It’s basically that you gonna pay for my time because I’m doing something for you and I’m not getting nothing in return for that.” – Age 25, unemployed.

“The guy that I was involved with, he didn’t want me to transition. And, he did everything he could. And he told me he was going to dump me. And, he did dump me. And I went and hopped in a relationship with another guy. And, I moved in with him the same day.” – Age 50, employed PT.

“Men feel like they can take advantage of a trans woman more than a real woman because it’ll be less consequences coming from a trans woman. I feel like there needs to be more people talking to trans women and letting them know to have your guard up. I just feel like it’s harder to get what we really want which is a relationship and just to be acknowledged and not feel like we’re so different. But the men make it that way.” – Age 21, employed PT.

## Discussion

This study sought to answer the question of how gender-affirming behaviors may enhance or diminish HIV vulnerability in light of structural and economic barriers to gender transition. Our results suggest that the process of gender transitioning for many trans women resulted in some aspects of HIV risk increasing while other aspects decreased both within and across individuals. As it relates to increasing HIV risk, our study found that the high need of trans women to have their gender affirmed by others contributed to trans women’s engagement in sexual risk behaviors, exposure to sexual objectification, and conflicting interests in HIV prevention messaging. For example, the intoxicating effects of drug use prior to sex are an established risk factor for unintended transmission of HIV [15, 16]. Yet, trans women in this sample described relying on non-prescribed drugs to be responsive to male sexual desires and to achieve their desired perceptions of feminine sexual behavior. Gender transitioning also required for some trans women to find new sex partners who they felt objectified trans women as sex workers and resulted in varying degrees of condom negotiation power. In addition, as some trans women gained an increasingly public transgender identity, destigmatizing trans women and other transgender communities became an important role. However, tensions in targeting HIV among transgender individuals resulted in some trans women being less receptive of HIV prevention messages, which may lead to lost opportunities to reach trans women. These findings point to an important need of developing non-stigmatizing prevention programs that enable trans women to be affirmed as women in low-risk sexual contexts. Addressing harmful pressures among trans women to conform to perceived feminine stereotypes is also important.

We additionally found that the combined effects of loss of housing and loss of employment due to gender transition along with the high cost of transition products (e.g., hormones, clothing) and clinical visits increased trans women’s reliance on sex work and created new obstacles in accessing HIV care. Studies have shown that sex work is associated with incident HIV and condomless anal sex [41]. Given that the process of coming out for some trans women resulted in being evicted from their homes by intimate partners or relatives, gender transitioning in the form of lost housing was attributed to engaging in sex work in new housing environments or having new co-habiting sex partner(s) with a new home. Loss of employment as well as trans women’s decisions to leave their workplace due to negative peer reactions was also attributed to sex work having an important economic role. Sex work also provided supplemental income to pay for desired affirming products and services. Findings also suggested that trans women with limited financial resources to pay for affirming services were further isolated from accessing or spending resources on HIV prevention. This suggests that policy initiatives involving financial assistance to trans women in the form of linkages to non-discriminatory employment, housing, and medical vouchers or insurance may play a vital role in reducing economic drivers of HIV risk during transition periods.

In contrast, our study also found that several gender-affirming behaviors were associated with lower HIV risk as trans women acquired medical and legal services relating to gender transitioning, redefined their sexual relationships, and received affirming support by others. For example, accessing medical services for transition for some trans women provided opportunity to test for HIV and other sexually transmitted infections (STIs) as well as obtain HIV/STI care and treatment. Trans women valued positive relationships and reported routine checks for HIV and transition services. This suggests that trans women whose current gender affirmation needs are met may be more likely to be exposed to and engage in HIV care. Medical transitioning for some women also involved a period of sexual abstinence due to a decrease in available sexual partners or bodily changes. While sexual abstinence was unwelcomed, it was described as a period of low risk to HIV. For others, obtaining a legal name change and having “passable” physical characteristics was viewed as lowering trans women’s risk for employment discrimination and enabling them to advocate for workers’ rights with non-sex work employers. Trans women with established transitions also described new roles in communicating with sex partners in promoting PrEP, taking advantage of free condoms, and selecting safer sex practices, such as oral sex. Lastly, gender-affirming behaviors by others, not just trans women themselves, were characterized as promoting well-being. For example, having trans-supportive landlords and trans-supportive employers protected against potential sexual assault at “male” shelters or workplace abuse. External affirming behaviors in the form of donated women’s clothing or transition guidance were also found to be important factors in overcoming transition barriers and protecting sexual health. Taken together, these findings suggest that an integrated strategy to reduce HIV should build upon expanded social, medical, and legal services for trans women that promote trans women’s individual efforts to protect themselves and that support sensitivity training of communities and service providers.

### Limitations

The results of this study should be interpreted in light of its limitations. First, our analysis relied on retrospective self-report which may have been influenced by recall bias. Some transition- or HIV-related factors may have been less noticeable or memorable. A second limitation is that given the cross-sectional design and relatively small sample size, we were unable to draw conclusions by sub-group, such as age or status. To characterize lifetime effects of transition, future studies may benefit from a larger sample or repeated interviews over time. In addition, despite our in-person and online recruitment efforts, the sub-sample of previously but not currently economically vulnerable trans women was small and limited our ability to characterize long-term economic successes. Lastly, although the study was advised by members of the sites’ transgender communities, including researchers who were members of the study population in the design and conduct of the study may have expanded our insights.

## Conclusion

This study used qualitative methods to examine how gender-affirming behaviors enhanced or diminished HIV vulnerability in light of structural and economic barriers to transition. Our study found that gender transitioning represents a complex period of empowerment and vulnerability which influences trans women’s resilience to protect against HIV infection, particularly in cases of economic hardship. More efforts are needed to support the sexual goals of individual trans women and implement structural and policy initiatives that promote safe gender transition and economic stability in U.S. trans women, particularly trans women of color. The combination of these interventions may play an important role in reducing HIV in this population.

## Data Availability

The data that support the findings of this study are qualitative transcripts and are therefore not publicly available due to their containing information that could compromise research participant privacy.

## Abbreviations

CAB: Community advisory board
CBO: Community-based organization
FT: Full-time employed
HIV: Human immunodeficiency virus
IRB: Institutional review board
LGBTQ: Lesbian, gay, bisexual, transgender, queer and/or questioning
MO: Missouri
MSM: Men who have sex with men
NGO: Non-governmental organization
PrEP: Pre-exposure prophylaxis
PT: Part-time employed
RA: Research assistant
STI: Sexually transmitted infection
TW: Trans women
UMSL: University of Missouri – St. Louis
US: United States
VA: Virginia
VCU: Virginia Commonwealth University
